# Evaluation of microleakage of class II dental composite resin restorations cured with LED or QTH dental curing light; Blind, Cluster Randomized, In vitro cross sectional study

**DOI:** 10.1186/1756-0500-7-416

**Published:** 2014-07-03

**Authors:** Faramarz Zakavi, Leila Golpasand Hagh, Soheila Sadeghian, Virginia Freckelton, Arash Daraeighadikolaei, Elham Ghanatir, Najmeh Zarnaghash

**Affiliations:** 1 Department of Operative and Esthetic Dentistry, Ahvaz Jundishapur Dental School, Ahvaz Jundishapur University of Medical Sciences, Ahvaz 6135715775, Iran; 2 Department of Periodontology, Ahvaz Jundishapur Dental School, Ahvaz 6135715775, Iran; 3 Department of Operative and Esthetic Dentistry, Urmia Dental School, Urmia 5714783734, Iran; 4 Department of Dental Practice, Oral Medicine Clinic, University of the Pacific Arthur A. Dugoni School of Dentistry, San Francisco, CA 94103, USA

**Keywords:** Class II cavity, Composite restoration, Microleakage, LED, QTH

## Abstract

**Background:**

The aim of this study is to compare the microleakage of Class II dental composite resin restorations which have been cured by three different LED (light emitting diode) light curing modes compared to control samples cured by QTH (quartz tungsten halogen) light curing units (LCUs), to determine the most effective light curing unit and mode of curing.

**Results:**

In this experimental study, class II cavities were prepared on 100 sound human premolars which have been extracted for orthodontic treatment. The teeth were randomly divided into four groups; three experimental and one control group of 25 teeth each. Experimental groups were cured by either conventional, pulse-delay, or ramped curing modes of LED. The control group was cured for 20 seconds by QTH. The restorations were thermocycled (1000 times, between 5 and 55°C, for 5 seconds dwell time), dyed, sectioned mesio-distally and viewed under stereo-microscope (40×) magnification. Teeth were then scored on a 0 to 4 scale based on the amount of microleakage. The data were analyzed by Chi-square test.

No significant difference was demonstrated between the different LCUs (light curing units), or modes of curing, at the enamel side (*p* > 0.05). At the dentin side, all modes of LED curing could significantly reduce microleakage (*p* < 0.05). The results suggest that slow start curing improves marginal integrity and seal. High intense curing endangers those aims.

**Conclusions:**

Comparison between the three LED mode cured composite resin restorations and QTH curing showed LED curing in all modes is more effective than QTH for reducing microleakage. Both LED and QTH almost completely eliminate the microleakage on the enamel side, however none of them absolutely eliminated microleakage on the dentin side.

## Background

Resin-based composites are synthetic resins which are used in dentistry for tooth restoration, or as an adhesive. There are two types of tooth colored restorative materials; self-cured and light-cured resins. The difference between them is the start polymerization, or how curing is accomplished. Mixing components is required in self-cured composites to begin polymerization. This may cause air-bubble porosity in the material. For light-cured resins, a hand held curing light that emits specific wavelengths works as the polymerization initiator.

In comparison with a common alternative restoration material, amalgam, synthetic resins exhibit superior aesthetics. High demand for tooth colored restorations in anterior and posterior teeth has triggered investigations of composite resin characteristics to improve poor outcomes such as microleakage which affects durability of tooth colored restorations, leading to restoration failure as well as post-operative sensitivity [1].

Thorough curing of composite resins is critical since it has a direct relation to the physical and mechanical characteristics of the resin composite. A main issue with composite resins is insufficient polymerization after curing. Insufficient polymerization due to poor curing, leads to increased water absorption, and compromised mechanical characteristics including less hardness, more erosion, micro-leakage, secondary caries, and as a consequence, failure of the composite filling [2,3]. One way to combat these problems is to select the best light curing system.

For many years, it has been common to use halogen light as the conventional curing mode [4]. Recently LED devices have come in to the dental market. Use of these devices is increasing due to durability of the device, no need to replace the filter and bulb, lower heat generation, and no cooling down [5]. In LED devices, there are three different modes for polymerizing and curing composite resins; ramped, pulse-delay, and conventional modes. Some researchers believe that there is no significant difference in restoration quality between the conventional curing technique with halogen devices, and LED curing, in terms of micro-leakage [6,7]. Other researchers have postulated that LED methods may not yield better results in reducing microleakage, in comparison to the conventional QTH curing method [8,9].

Considering the increased use of LED devices, and also considering contradictions in previous research results, the current study, comparing the effects of three different methods of LED composite resin curing with conventional QTH curing is timely. This will clarify evidence based outcomes to help dental practitioners choose the most valid and reliable method of light curing. In the current study our main question is whether replacing a QTH curing unit with a LED one, and applying one of these different methods of LED curing will reduce, increase, or cause no significant effect in terms of micro-leakage in enamel or in dentin in comparison to QTH composite resin curing. And we have tested the hypothesis of “micro-leakage of class II dental composite restorations with QTH dental curing light is higher than LED curing produces”.

## Methods

This in vitro study is a double blind, imbalanced randomized (3:1), cross sectional study conducted in Iran. In this experimental in vitro study, we used 100 intact human premolars, free of caries and fracture lines, which have been extracted already for orthodontic treatment, and we did not extract any tooth in order to perform this study. The study was carried out in the restorative laboratory of Ahvaz Jundishapur School of Dentistry, Ahvaz, Iran, from June 2011 to July 2012. This study was approved by the Research Ethics Committee of Ahvaz Jundishapur School of Dentitry (U-90233). Any experimental research that is reported in this manuscript, have been performed with the approval of an appropriate ethics committee.

**Figure 1:**
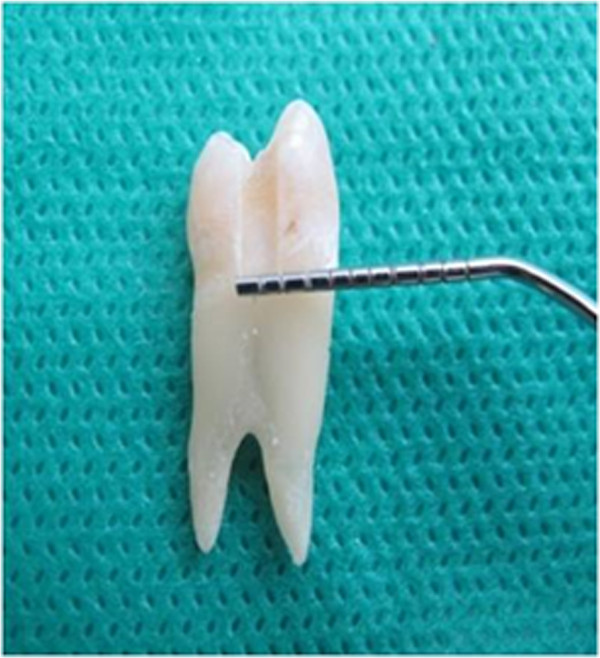
Cavity dimensions.

**Figure 2:**
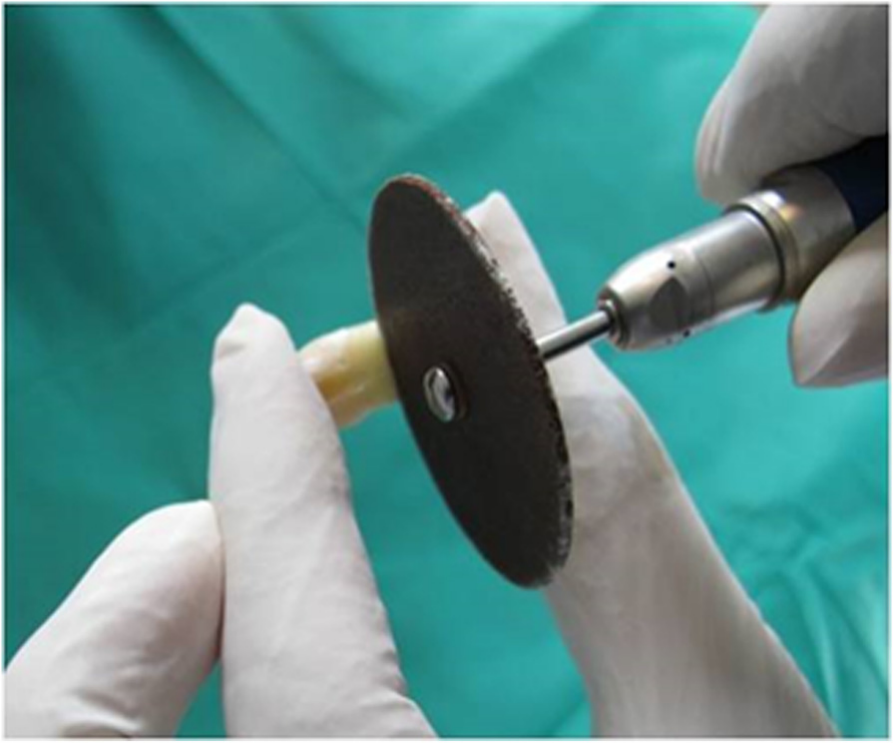
Cutting the root tip.

**Figure 3:**
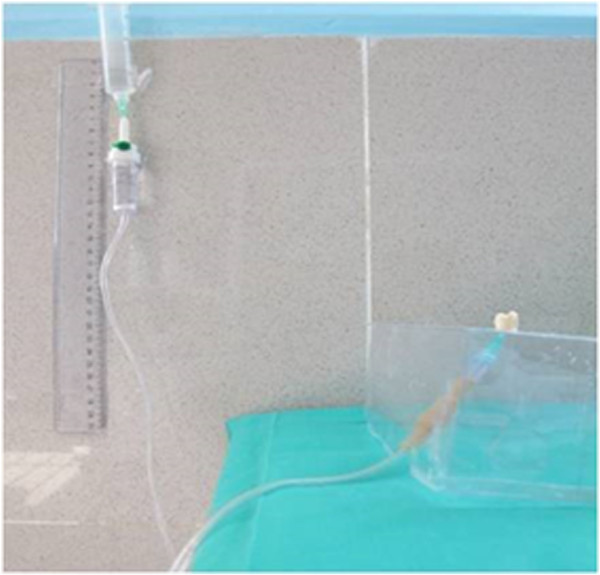
Pulp pressure simulation environment.

The teeth were rinsed with a soft brush and water, and maintained in normal saline solution. A restorative specialist prepared similar size class II cavities with the occluso-gingival and mesio-distal dimensions of 2 mm and bucco-lingual dimension of 1.5 mm (Figure 1), all with the same high speed handpiece (PAXTUB2, NSK, Shinagawa, Tokyo, Japan), diamond bur (835KR010, D&Z, Goerzallee, Berlin, Germany), and cooling system for air and water, to avoid confounding factors. The gingival margin was prepared 1 mm lower than CEJ. The apical half of each root was separated by a diamond disk (Figure 2). The pulp was exposed, and pulp tissue removed by a small barbed broached file. The pulp canal was irrigated with a 2.5% sodium hypochlorite solution for 30 seconds and the tooth was kept in water for 30 minutes. All coronal segments were attached to a plate, using sticky wax. An 18 gauge stainless steel tube was inserted into the center of this plate. The entry of the stainless steel tube to the pulp chamber was sealed with sticky wax. The tube was connected to a 10 cm plastic syringe through a plastic pipe. To simulate the vital tooth pulp pressure of 34-40 cm H_2_O (Figure 3) in all samples, a syringe reservoir, filled with distilled water, was placed 25 cm above the tooth surface to create the pressure of 35 cm H_2_O at the dentin surface. The same pressure was used for all cases.

**Figure 4:**
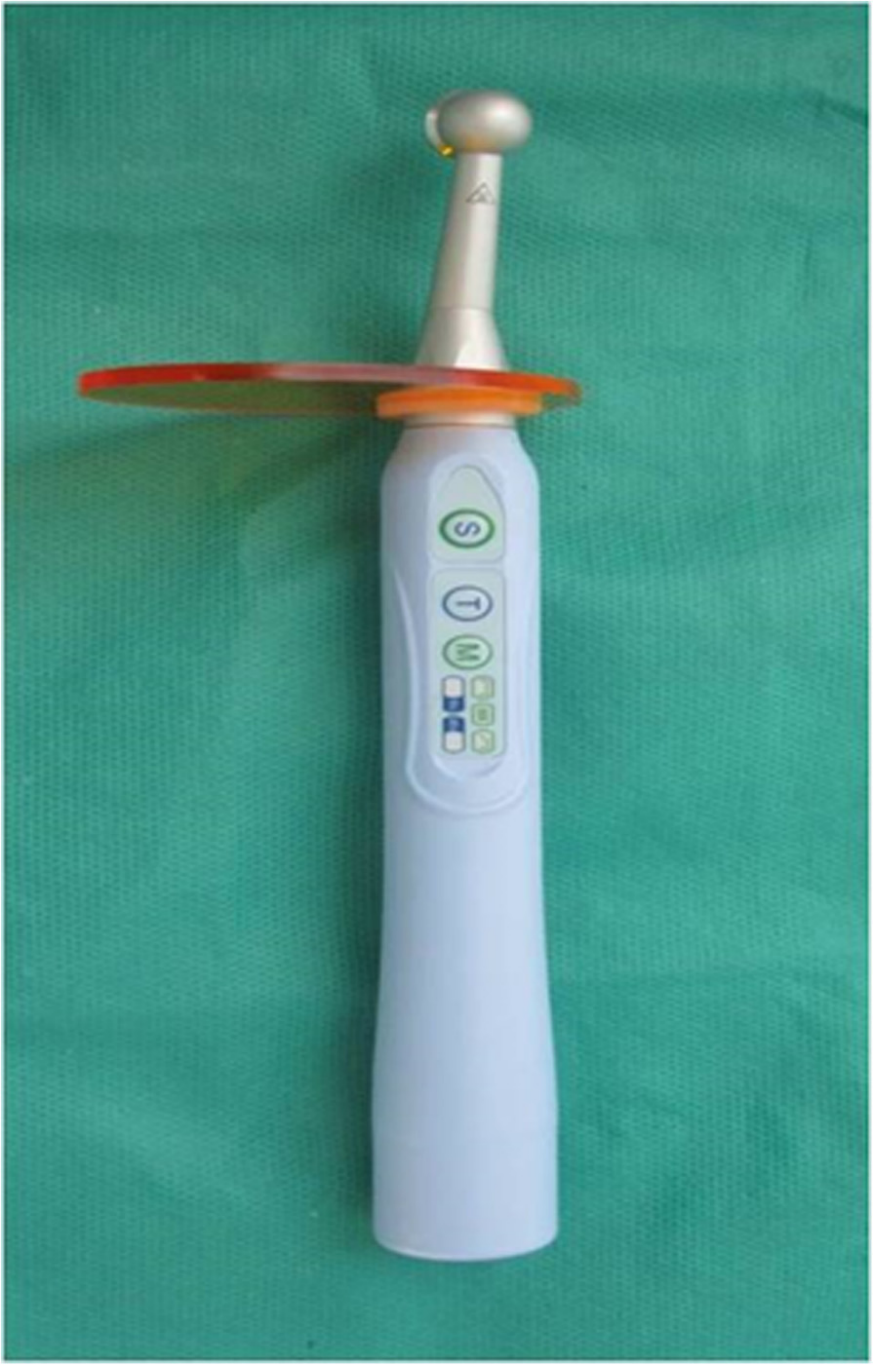
LED curing unit.

**Figure 5:**
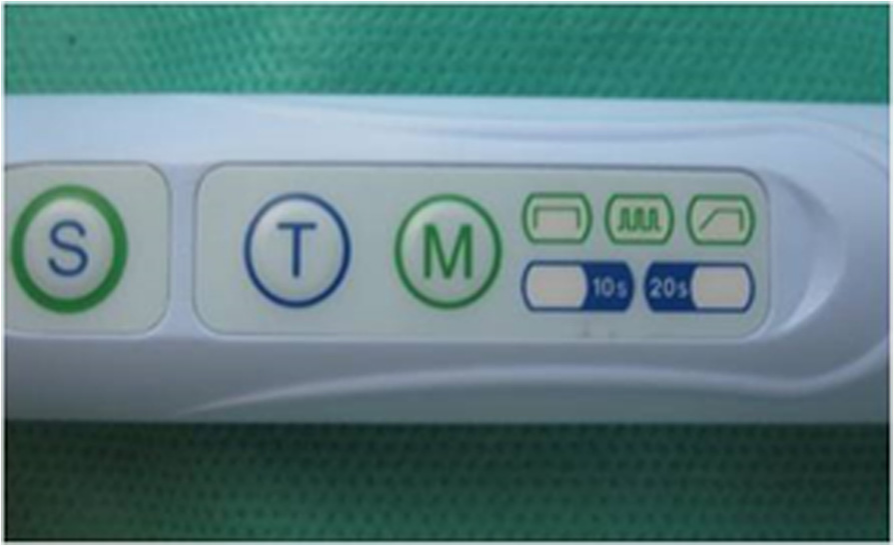
LED different curing options.

**Figure 6:**
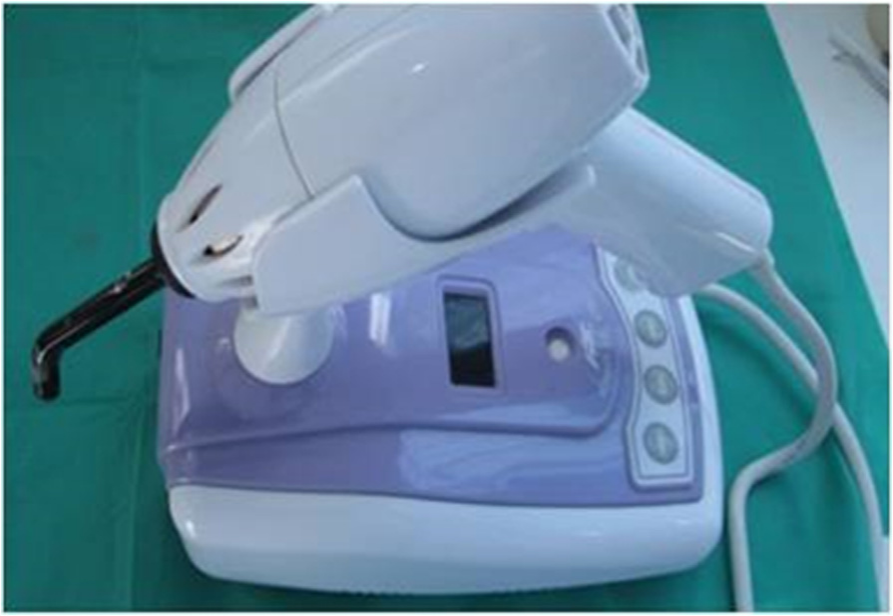
QTH curing unit.

While all teeth were connected to the syringes for pulp pressure simulation, the cavity preparations were etched for 15 seconds with 35% phosphoric acid gel (Ultra-Etch, Ultradent, South Jordan, UT, USA) and rinsed for 10 seconds. After applying a single bond (3M ESPE, St ovePaul, MN, USA), the cavities were filled with the micro hybrid composite (A1 colour) Valux (3M ESPE, St Paul, MN, USA). Afterward, the entire surfaces of the fillings were polished with Sof-Lex™ (3M ESPE, St. Paul, MN, USA) finishing disks.Teeth were randomly divided into 4 groups of 25 (three experimental groups and one control group). Experimental groups were exposed for 20 seconds to one of the three modes conventional, pulse-delay, ramped) of LED light (Bonart, ART-L3, Xinzhuang District, New Taipei City, Taiwan) “(Figures 4 and 5)”. The control group was cured for 20 seconds by QTH (Figure 6, Bonart, ART-L2, Xinzhuang District, New Taipei City, Taiwan).

### The different modes of LED composite curing were applied as follows

#### Conventional

Irradiation started at the highest intensity (400 mw/cm2), continues without alteration in intensity to the end curing time. The output radiation wavelength remained 450-470 nm.

#### Ramped

Irradiation was initiated at 100 (400 mw/cm2) increasing to maximum intensity which was maintained to the end of the curing time.

#### Pulse

Irradiation started with 400 (400 mw/cm2) for 1 second, then 0.2 delay. This output was repeated to the end curing time.

### QTH curing mode was applied as follows

**Figure 7:**
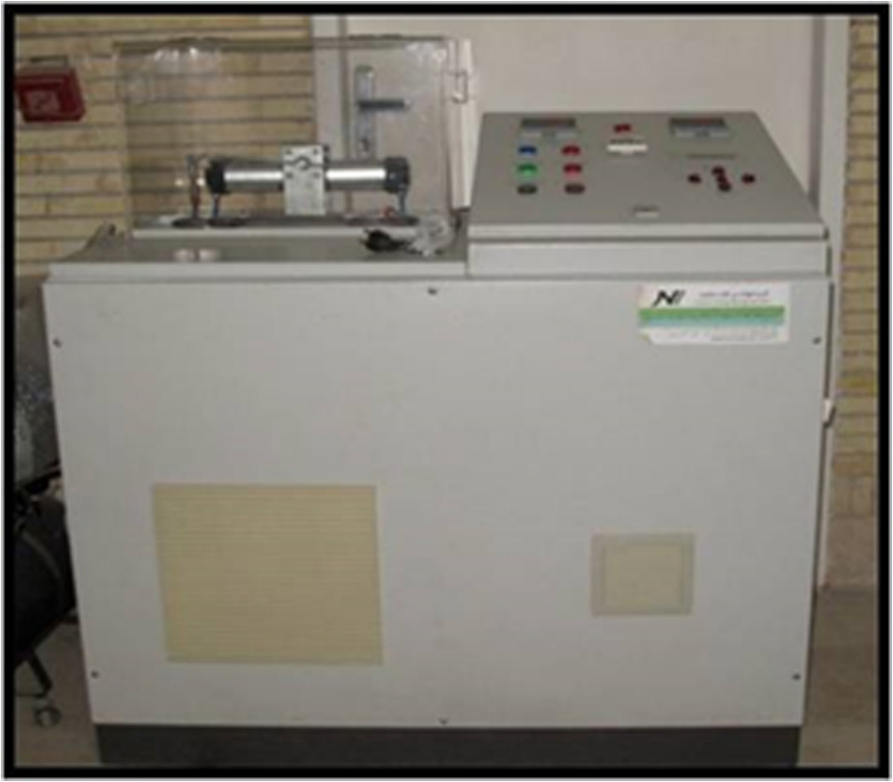
Thermocycling unit.

**Figure 8:**
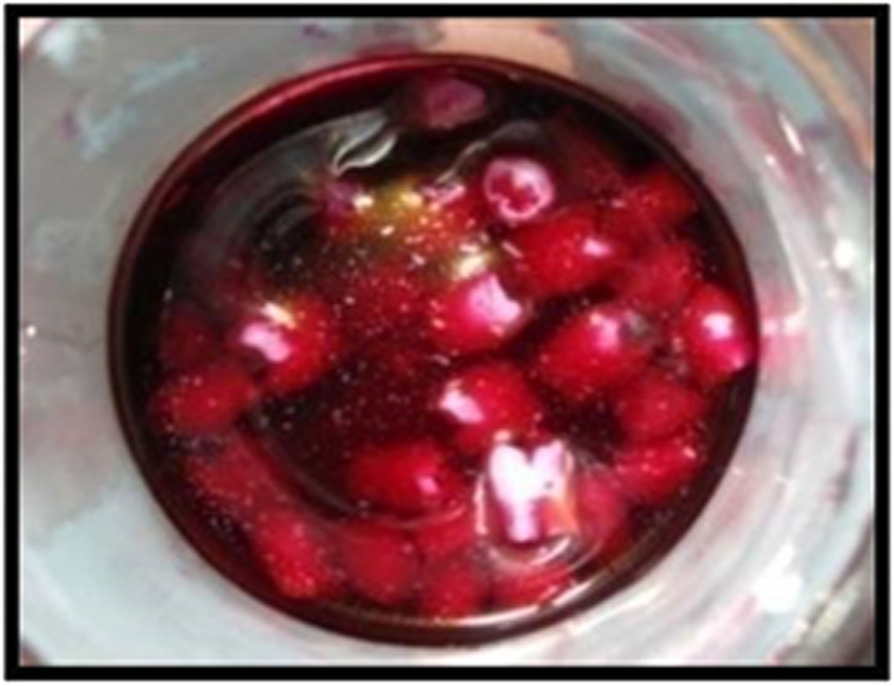
All teeth were immersed in 2% basic fuchsine for 24 hours.

**Figure 9:**
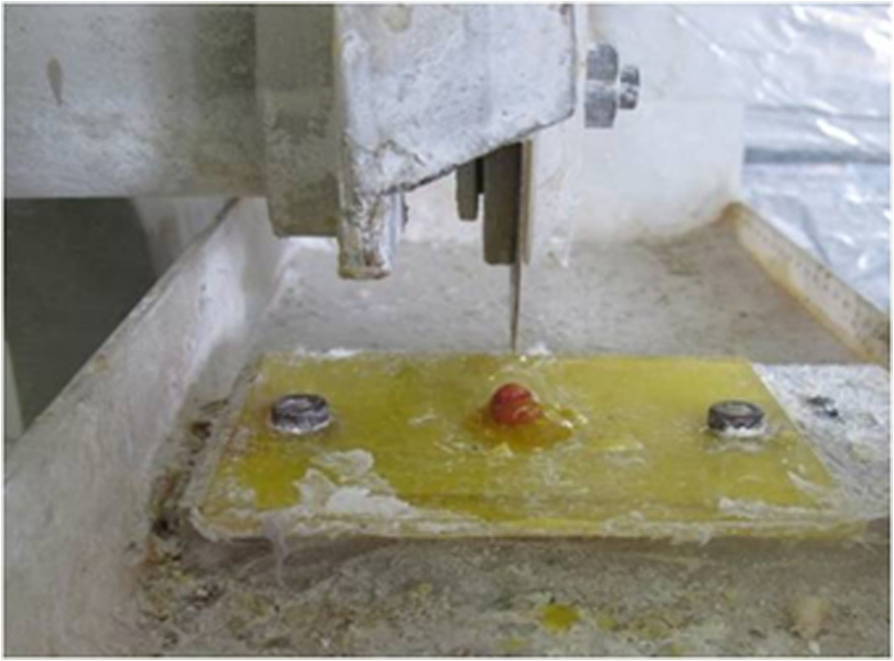
Section unit diamond disk.

**Figure 10:**
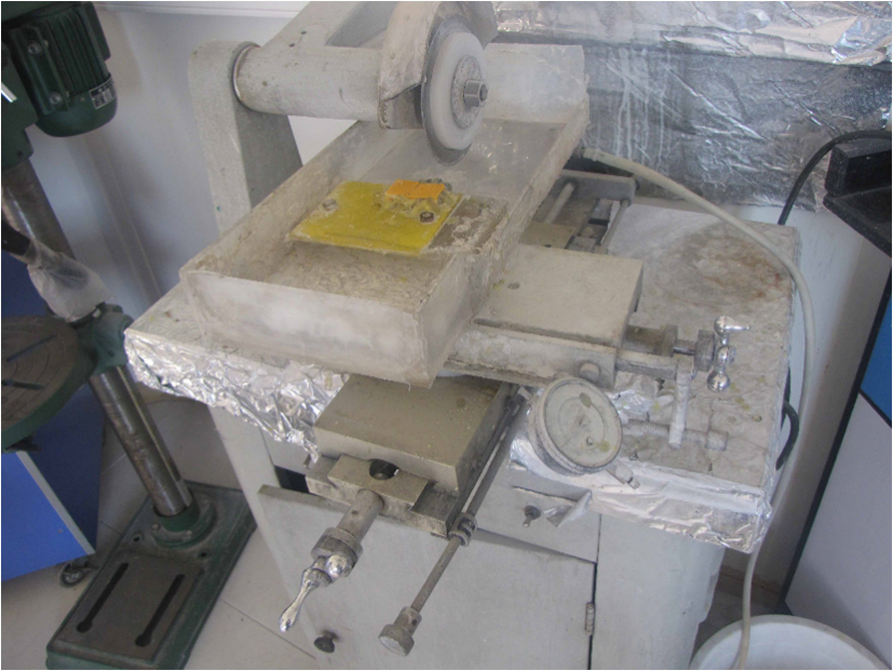
Section unit.

**Figure 11:**
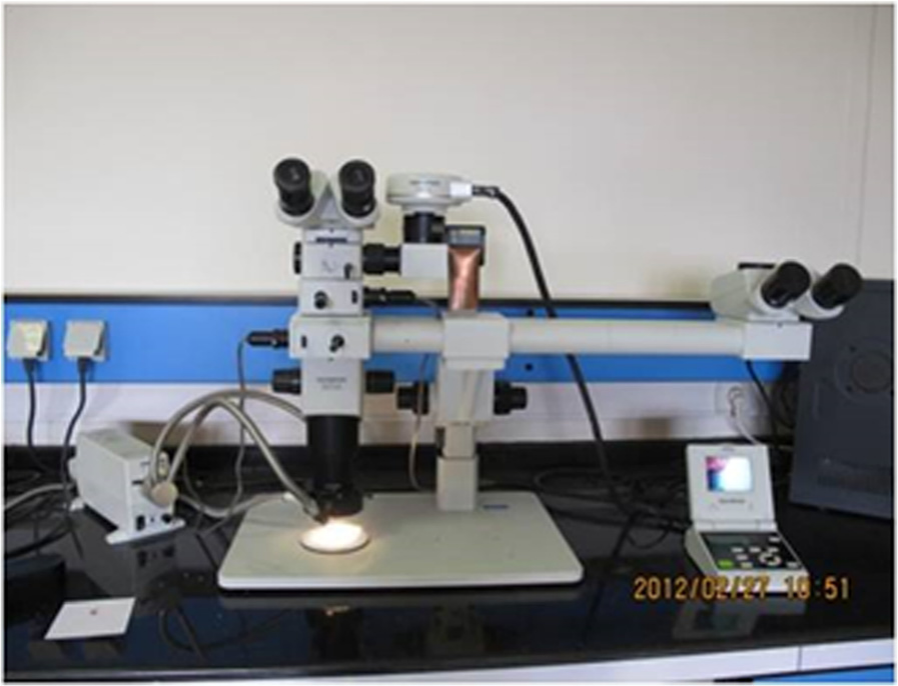
Stereo-microscope unit.

**Figure 12:**
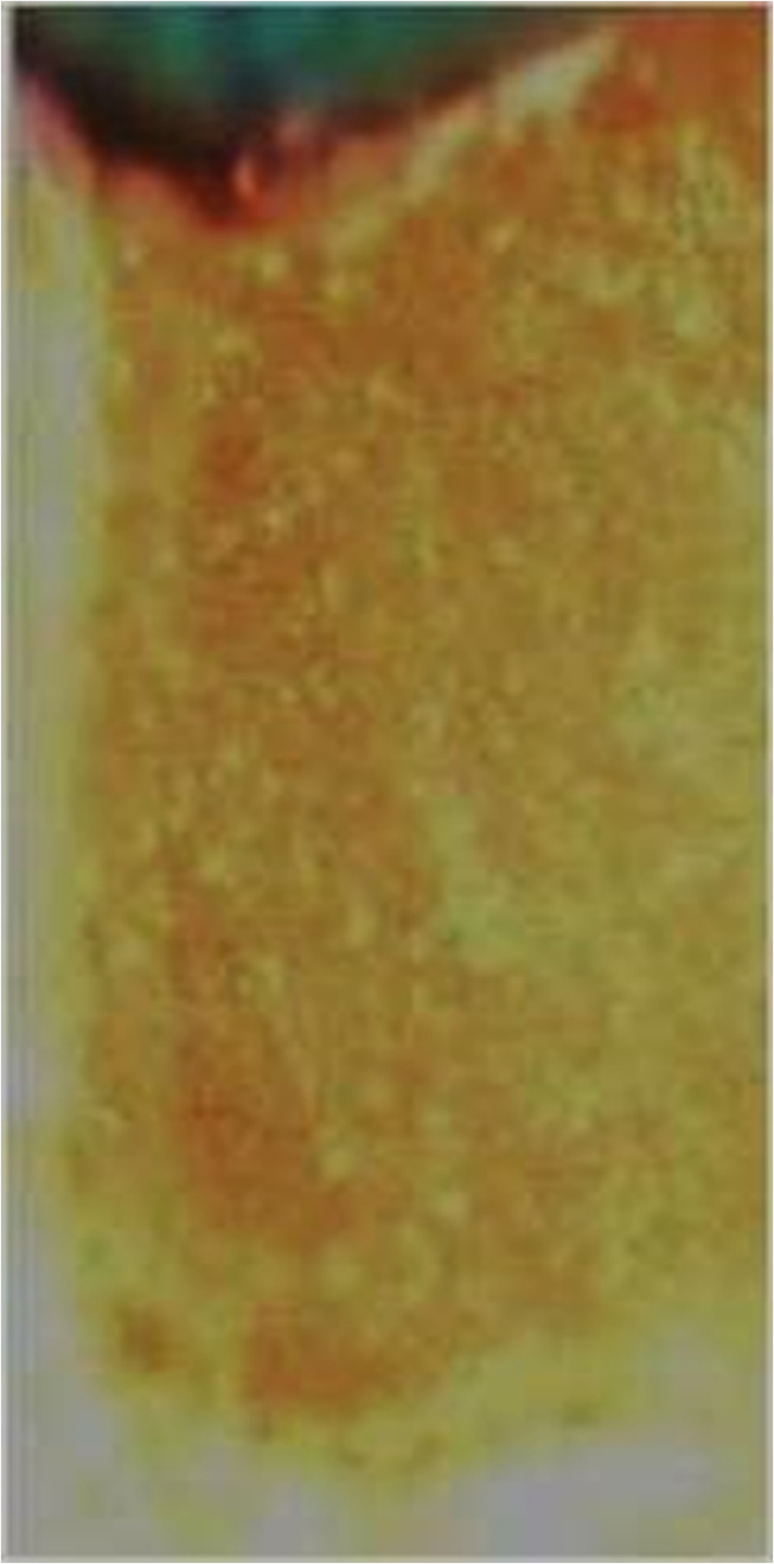
Score 0 of fillings under stereo-microscope.

**Figure 13:**
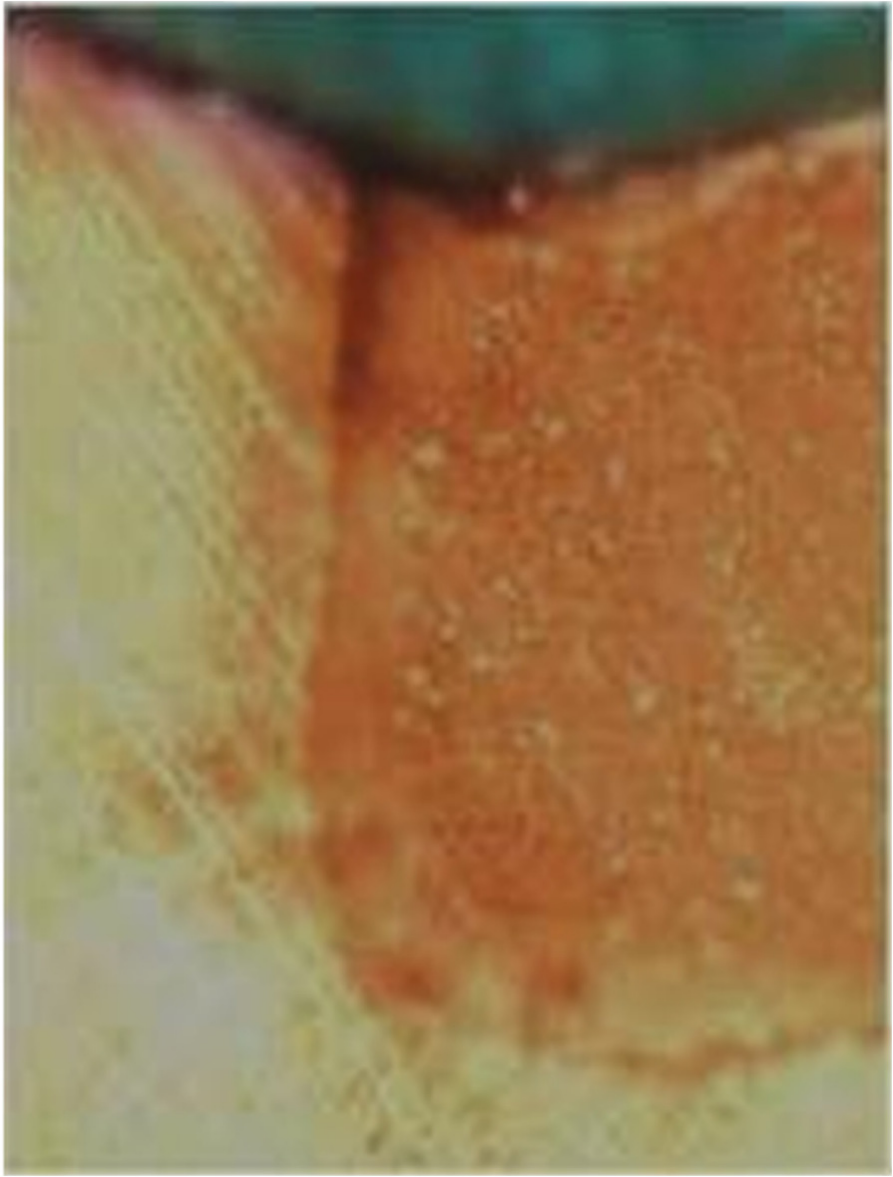
Score 1 of fillings under stereo-microscope.

**Figure 14:**
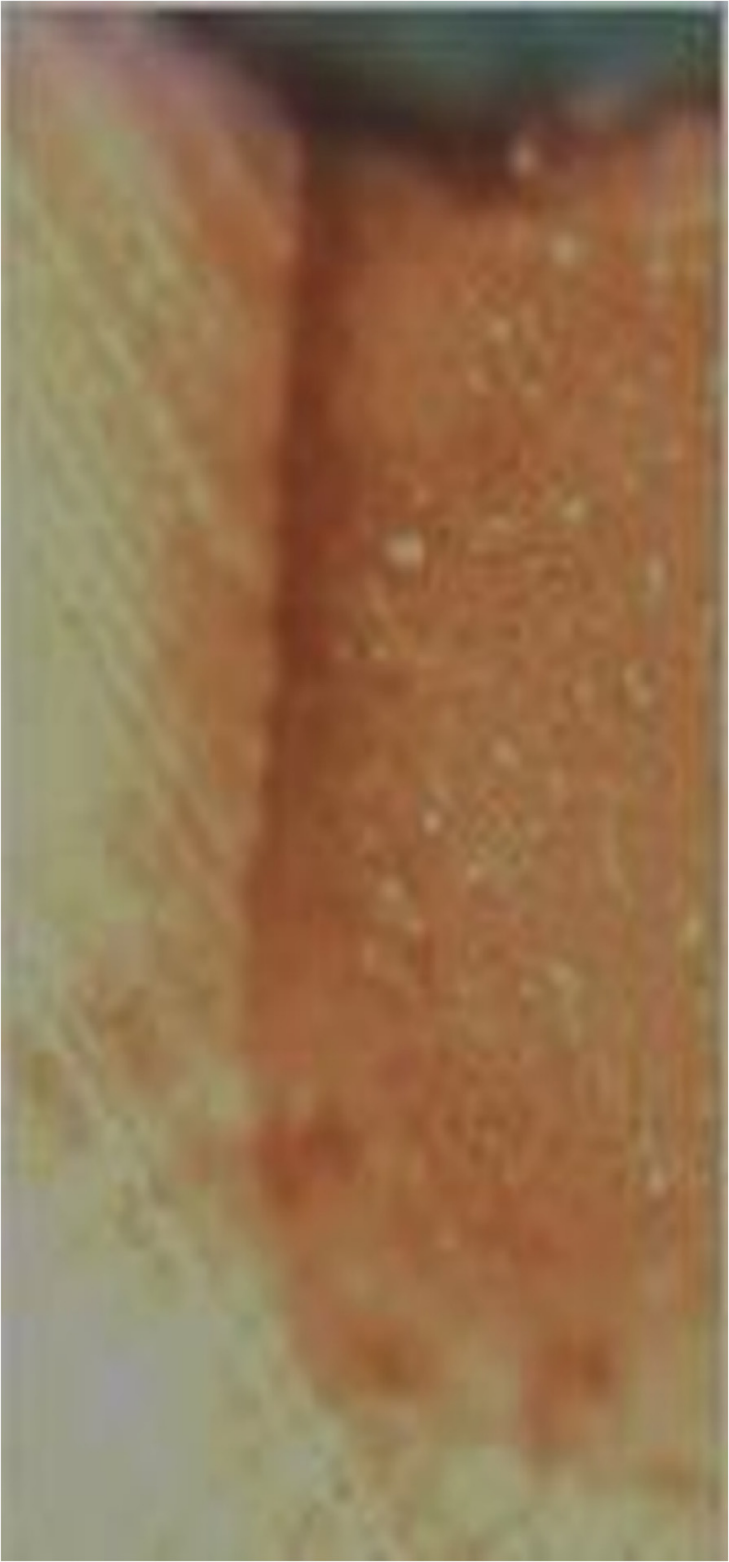
Score 2 of fillings under stereo-microscope.

**Figure 15:**
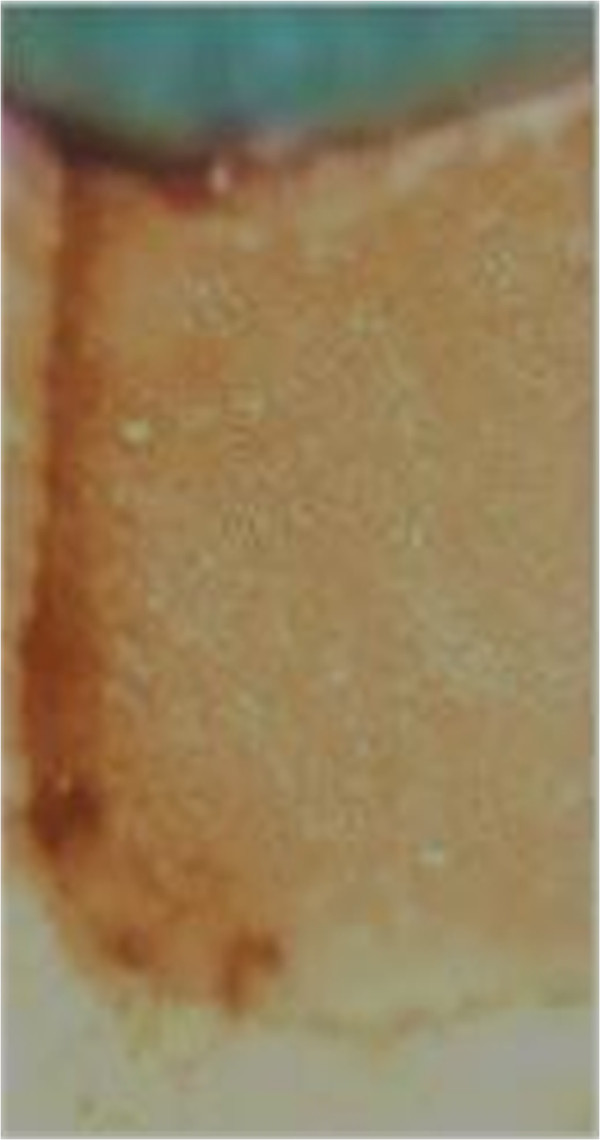
Score 3 of fillings under stereo-microscope.

**Figure 16:**
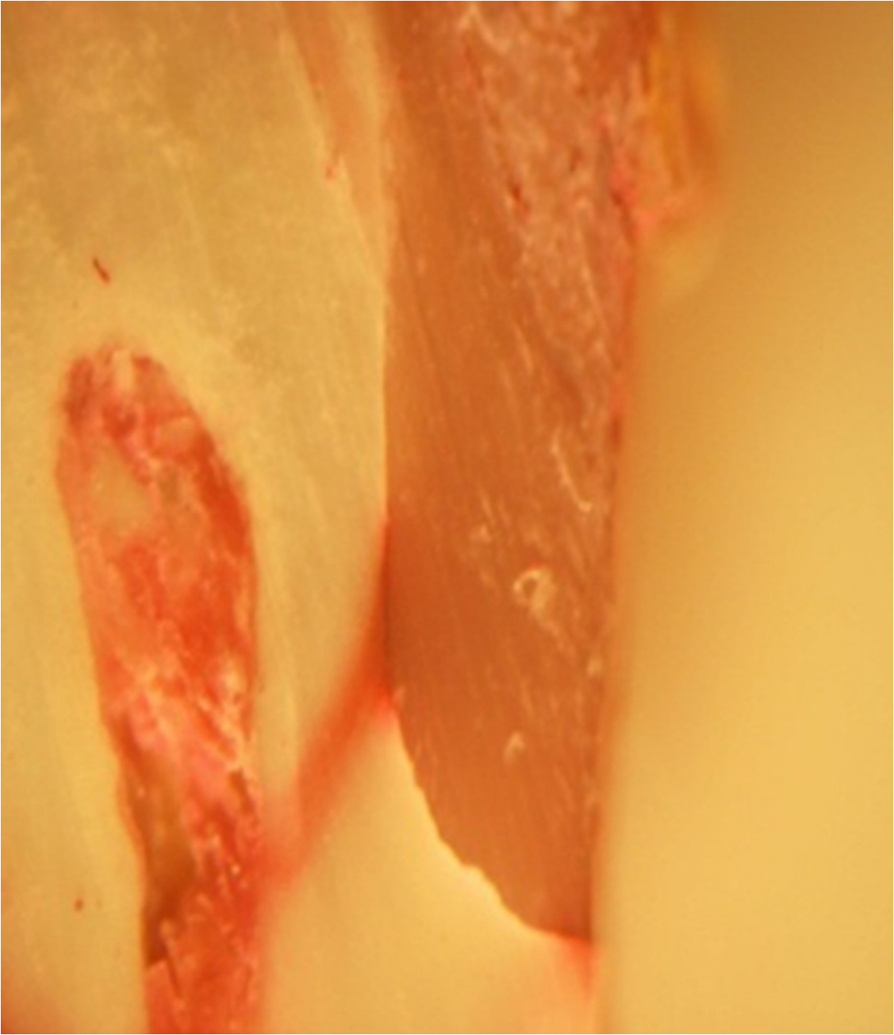
Score 4 of fillings under stereo-microscope.

The output radiation intensity of the QTH resin composite curing method was between 400-500 mw/cm2.All surfaces of the teeth were sealed up to a 1 mm border around each preparation margin with nail polish. When the nail polish dried, the root ends were sealed by the sticky wax. Then the teeth were thermo-cycled (Figure 7, Thermo-cycling Machine, 86-1, Naftmachine Group Engineering, Tehran, Iran) for 1000 cycles between 5°C and 55°C, using a dwell time of 5 seconds. All teeth were immersed in 2% fuchsin basic dye (Figure 8, Certistain Fuchsin, CI 42510, Merck, KGaA, Darmstadt, Germany) for 24 hours, and then were rinsed under water.A longitudinal section was made; Figures 9 and 10 by sectioning machine (Gilling-Hamco, Thin Sectioning Machine, Hamco Machines, Inc, Rochester, New York, USA) with a diamond disk (FEJ, Germany) through each filling in a mesio-distal plane, then viewed under a stereo-microscope unit (Figure 11, Olympus, SZX9, Tokyo, Japan) at ×40 magnification; (Figures 12, 13, 14, 15 and 16).

All materials which have been used in this study are shown in the first three tables by their brand name, model, name of the company, city, province, and country. Microleakage has been assessed based on dye penetration, through a common scoring method of 0-4 per the following criteria:

1: Dye penetration less than half the length of the gingival floor,

2: Dye penetration greater than half, up to the whole length of the gingival floor,

3: Dye penetration the whole length of the gingival floor plus up to half of the axial wall.

4: Dye penetration the whole length of the gingival floor plus greater than half the axial wall and existence of lateral microleakage at dentin tubules.

A successful reading displayed evidence of one of the designated categories, an unsuccessful test was not readable.

One hundred intact human premolar teeth were collected, stored, disinfected and handled per the guidelines of OSHA and the CDC. Sample size calculations were performed based on standard deviation and statistical error estimates. For assigning the teeth into four groups, a computer-generated list of random numbers was used. Researchers (both senior and junior) were blind to which teeth were randomly divided into three experimental groups and one control group. The data were analyzed using the Chi- square test.

## Results

The result of this study provides useful clinical information for effective selection of curing lights and curing modes to reduce microleakage in composite resin dental restorations.

### Enamel

Most score 0 was reported for specimens in all groups. The Chi-square test, (*p* = 0.06% (*p* > 0.05%) achieved), shows no statistical difference among the four groups (Table 1).

**Table 1:** Microleakage in enamel

**Total**	**1**		**0**		**Score**
		**Percentage**	**Numbers**	**Percentage**	**Numbers**	**Group**
25	32	8	68	17	QTH
25	20	5	80	20	LED conventional
25	12	3	88	22	LED pulse-delay
25	24	6	76	19	LED ramped
P-value = 0.06					

### Dentin

Most score 4 was reported for specimens of QTH and conventional cure LED groups.

The highest numbers of score 3 went to LED pulse-delay = 56% and LED ramped = 52% specimens. Since the result of the Chi-square test is *p* = 0% (*p* < 0.05%), there is a statistical significant difference among the groups. Therefore the binary comparisons of the groups were studied (Table 2).

**Table 2:** Microleakage in Dentin

**Total numbers of successful tests (excluding non-readable)**	**Total number of samples**	**4**		**3**		**2**		**1**		**Score**
		**Numbers**	**Percentage**	**Numbers**	**Percentage**	**Numbers**	**Percentage**	**Numbers**	**Percentage**	**Group**
24	25	19	76	1	4	3	12	1	4	QTH
24	25	23	92	0	0	1	4	0	0	LED conventional
25	25	0	0	14	56	11	44	0	0	LED pulse-delay
24	25	2	8	13	52	9	36	0	0	LED ramped
P-value = 0.00										

Based on the Chi-square tests, the binary comparisons of all pairs of groups were statistically significant, except for the comparison of LED (pulse delay) vs. LED (ramped) groups.

## Discussion

Because enamel structure is homogenous, adherence to the enamel is expected to be dependable and readily achievable. However, acceptable adherence to the dentin is more complicated, due to factors including being non-uniform, movement of dentin liquid toward the external surface of dentin, and lesser percentage of organic compounds [10]. If the shrinkage force of polymerization is higher than the initial adherence strength of the composite to the dentin, a gap will occur. This phenomena happens more often in root surfaces, as opposed to the crowns of teeth [1]. In this study, most samples did not show microleakage at the enamel margins, which was in agreement with other studies [10-15].

Based upon this study, microleakage in dentin using QTH and LED (conventional curing mode), were often scored 4, while LED (ramped and pulse-delay modes) were scored 3, indicating less microleakage. This study suggests that slow-start light irradiation improves seal and marginal integrity; however unmodulated intense irradiation compromises this goal, correlating with previous research results [11,12,16].

According to the findings of this research, the microleakage detected in QTH light cured samples, is significantly higher than in samples cured by three modes of LED. Potentially this may be due to the coordination of LED optical output wavelength with the comphorquinone absorption spectrum (the photo-initiator of conventional resin composites). The amount of microleakage in the modes of ramped and pulse-delay curing by LED device was similar. As described earlier, the amount of microleakage appeared to be associated with the intensity of the LED light source. Unmodulated light intensity appears to cause an increase in polymerization shrinkage and therefore more microleakage, in this study.

LED light cure releases well-defined stimulated electrons. The output spectrum of LED is coordinated with the absorption spectrum of comphorquinone photo-initiator (450-500 nm), therefore it does not need any filter. It has been postulated that two possible methods to reduce polymerization shrinkage stress reduction are soft-start curing and pulse delay, which have been confirmed in this study. However some studies indicate that the amount of dentinal microleakage in both LED light cure device, and QTH, is similar. It may be due to the different intensity of various applied light curing units [1,13,14,17-19].

Bouschlicher *et al.*[19], reported similar evidence of polymerization shrinkage comparing a stepped light curing method and unmodulated QTH curing, which contradicts our results. They applied 100 mw/cm^2^ initial light intensity for 10 seconds and 800 mw/cm^2^ final intensity for 30 seconds. Their higher final intensity was higher than our method, which may be a reasonable justification for the higher shrinkage and microleakage.

In one investigation, microleakage between LED and QTH cured composite resin samples after 3 months storage time was not statistically significant, whereas in a 24 hours period there was meaningful leakage noted [12]. Water absorption of composite is inevitable resulting in hygroscopic expansion. Volumetric expansion compensates for the initial shrinkage and temporarily reduces microleakage. This expansion occurs in first few days. After one week, when the resin composite is saturated, the compensating effect stops and microleakage increases due to resin composite solubility [2].

Hassani Tabatabaei *et al.*[10], in opposition to the results of our study, reported that in dentinal walls, microleakage produced by LED soft-start curing and LED pulse-delay methods is higher than in the QTH conventional curing method (Astralis 7 Ivoclar Vivadent QTH). Their findings are based upon a different composite resin choice, and a higher light intensity LED unit; comparison with our findings is difficult.

Frield K. *et al.*[20] and Hofmann N. *et al.*[21] have reported results which conflict with the present study results. They have used higher light intensity (more than 600mw/cm^2^) in their research, while in our study, the light intensity was gradually increased and reached up to 400 mw/cm^2^ at the peak. Our results suggest the advantages of lower initial polymerization: Higher elasticity, and lower tension in the cured material produced by the soft-start method may be reduced if the final exposure is too high, causing an increase in total shrinkage.

## Conclusions

Results of this study indicate that there is no significant difference among various methods of LED curing, and QTH curing, at the enamel margins reflected by score 0 for most specimens. However, at dentin margins, there are significant differences.

Both LED and QTH eliminate microleakage almost completely at the enamel margin; however none of them can absolutely overcome the dentinal microleakage. Between two light sources (LED and QTH), LED is observed to be more effective in all different modes. LED composite resin curing offers better penetration depth, lower heat production, and repeatable clinical results in comparison to QTH light curing. Delay-pulse and ramped LED curing reduced dentinal microleakage in a statistically significant manner compared to unmodulated LED and QTH curing lights (Table 3).

**Table 3:** Conclusion table

	**QTH**	**LED (Conventional)**	**LED (pulse delay)**	**LED (ramped)**
QTH	-	S	S	S
LED (Conventional)	S	-	S	S
LED (pulse delay)	S	S	-	NS
LED (ramped)	S	S	NS	-

## Competing interests

The authors declare that they have no competing interests.

## Authors’ contributions

FZ designed the study, carried out cavity preparations, and performed the statistical analysis. LGH conceived the study and helped to draft the manuscript. SS performed the laboratory assays and helped to draft the manuscript. VF performed the final statistics and rewrote the final manuscript, AD made substantial contributions to conception, design, acquisition of data, the analysis and interpretation of the data, and was involved in drafting and reviewing the manuscript for important intellectual content. EG helped to collect the data. NZ helped to collect the data. All authors contributed to the study and confirmed the final version of the submitted manuscript. All authors read and approved the final manuscript.

## Authors’ information

FZ is the dean of Ahvaz Jundishapur Dental School and head of restorative and operative department.
